# Case report: Disseminated intravascular coagulation in a dog following treatment with melarsomine for *Dirofilaria immitis*

**DOI:** 10.3389/fvets.2023.1118798

**Published:** 2023-02-06

**Authors:** Helen S. Philp, Kate S. Farrell, Ronald H. L. Li

**Affiliations:** ^1^William R. Pritchard Veterinary Medical Teaching Hospital, University of California, Davis, Davis, CA, United States; ^2^Department of Veterinary Surgical and Radiological Sciences, School of Veterinary Medicine, University of California, Davis, Davis, CA, United States

**Keywords:** adulticide, heartworm, hemorrhage, melarsomine, coagulopathy

## Abstract

Disseminated intravascular coagulation following melarsomine therapy for *Dirofilaria immitis (D. immitis)* is reported in a 9-year-old female intact pit bull-type dog. The dog had been diagnosed with *D. immitis* (antigen and microfilaria positive) and treated with imidacloprid, moxidectin, doxycycline and 3 doses of melarsomine over a 92-day period. Seven days after the third melarsomine injection, the patient was presented to her family veterinarian due to right pelvic limb swelling. Prothrombin and activated partial thromboplastin times were prolonged beyond the detectable range. Treatment included vitamin K1 and fresh frozen plasma (FFP) prior to referral to the authors' institution. At this time the patient remained coagulopathic. Further investigations included thoracic radiographs, abdominal ultrasound and an echocardiogram. The patient was administered multiple units of packed red blood cells and FFP, sildenafil, dexamethasone SP, aminocaproic acid and vitamin K1. Repeat CBC approximately 20 h after admission showed persistent anemia and thrombocytopenia. Despite ongoing administration of FFP, a repeat coagulation panel showed worsening of the coagulopathy with prothrombin time of 84.2s [reference interval (RI) 7.0–9.3s], activated partial thromboplastin time >140s (RI 10.4–12.9s) and fibrinogen <50 mg/dL (RI 109–311 mg/dL). Following discussion with the owners, the patient was euthanized. Necropsy was performed and confirmed heartworm infection with severe pulmonary arterial thrombosis, vascular remodeling, and intraluminal degenerate nematodes. Multifocal subcutaneous and organ hemorrhage was apparent. Although coagulopathy has been described in caval syndrome associated with heartworm disease and is listed as a potential side effect of melarsomine administration, this is the first report of documented disseminated intravascular coagulation following melarsomine treatment for *D. immitis*. Potential mechanisms for the coagulopathy are discussed and the case report highlights a rare, but serious complication of adulticide therapy.

## 1. Introduction

*Dirofilaria immitis* is a parasitic filarial nematode responsible for heartworm disease in dogs, which are the definitive host (1). It has a long and complex life cycle involving transmission by a mosquito vector and several molts prior to sexual maturity (2). *D. immitis* also harbors *Wolbachia*, a gram-negative bacterium which is required for its reproduction (3). Mature heartworms mainly reside in the caudal pulmonary arteries and may cause physical obstruction, inflammation, and endothelial dysfunction. *Wolbachia* appears to contribute to immune polarization during infection as well as inflammatory/immunomodulatory activity *via* surface protein antigens (4). Clinical signs (if any) depend on the severity of disease and duration of infection (3).

In order to eliminate all life stages of the heartworms while minimizing complications, the American Heartworm Society (AHS) has published a detailed management protocol (5). As part of this protocol, adulticide therapy with melarsomine is recommended (5). In many, if not most cases of canine heartworm infection, melarsomine is considered a safe and effective adulticide (5). However, worm death commonly leads to some degree of thromboembolic disease and a number of clinical sequelae have been described (3). Treated dogs must be carefully monitored for adverse cardiopulmonary effects and intense inflammatory reactions (6).

Disseminated intravascular coagulation (DIC) is a recognized complication in heartworm-infected dogs with caval syndrome (7). DIC has also been reported clinically in heartworm-infected dogs following treatment with thiacetarsamide (8, 9) (the predecessor to melarsomine) and with dichlorvos (10). DIC is also listed as a potential complication (occurring in < 1.5%) of dogs treated with melarsomine dihydrochloride on the drug data sheet (11). However, the authors were unable to find any published data on melarsomine-induced coagulopathy or DIC post-treatment with melarsomine in dogs.

The purpose of this report is to describe the diagnosis and treatment of a dog with DIC following melarsomine treatment for *D. immitis* and to draw attention to a rare but potentially fatal complication of adulticide therapy.

## 2. Case description

An estimated 9-year-old female intact pit bull-type dog was presented to a veterinary teaching hospital for management of coagulopathy. The dog was initially adopted from a shelter 4 months earlier. At that time, she had seemed to be in good health except for some intermittent coughing. CBC and serum chemistry during a wellness check were unremarkable. Thoracic radiographs performed at that time showed a mild bronchointerstitial pulmonary pattern. A small mass on the ventral abdomen was resected and histopathology identified a low-grade cutaneous hemangiosarcoma (HSA) with mild solar elastosis. Complete excision was confirmed. The patient was presented again 3 days later for a recheck and vaccinations. A point-of-care bidirectional flow ELISA^1^ test was positive for *D. immitis* antigen.

Following confirmation of microfilaremia by a modified Knott test, imidacloprid and moxidectin (Advantage Multi^2^) was administered (Day 0). The patient was started on doxycycline^3^ at 10.8 mg/kg PO every 12 h which was continued for 5 weeks at which time another dose of imidacloprid and moxidectin was administered. On Day 59, another dose of imidacloprid and moxidectin was applied and the patient was started on a tapering dose of prednisone^4^ (0.5 mg/kg PO q 12 hrs for 1 week, 0.5 mg/kg PO q 24 hrs for 1 week and then 0.5 mg/kg PO every other day for a further 2 weeks). On Day 60, a 2.5 mg/kg dose of melarsomine^5^ was administered intramuscularly (IM). On Day 91, a second dose of melarsomine was administered IM and a similar tapering course of prednisone dispensed. Imidacloprid and moxidectin was also applied. One day later (Day 92), a third dose of melarsomine was administered IM. Owner history revealed that the dog had been lethargic for 1–2 days after the first melarsomine injection but had returned to normal after this. After the second and third doses of melarsomine, the patient had become lethargic again and was shivering. Since then, she had remained lethargic and inappetant. The patient had been exercise restricted since administration of the third dose of melarsomine (cage rest and short leash walks for toileting).

Seven days after the third melarsomine injection, the patient was presented to her family veterinarian due to right pelvic limb swelling. Some gingival bleeding was also noted. Complete blood count revealed a non-regenerative normocytic, normochromic anemia with a HCT of 0.247 L/L (24.7%; RI 0.387–0.592 L/L [38.7–59.2%]). Thoracic, abdominal and right pelvic limb radiographs were performed and interpreted by a board-certified radiologist. The cardiopulmonary structures and abdomen were considered normal. The superficial soft tissues of the distal right pelvic limb were mildly swollen consistent with edema or cellulitis. The patient was transferred to an emergency facility for ongoing care. On arrival the patient was bright and alert. Several ecchymoses and subcutaneous swellings were noted in addition to swelling of the right hock and crus.

The patient was hospitalized and started on supportive care. Some evidence of active hemorrhage was noted with swelling at venipuncture sites, cutaneous bleeding, and progression of ecchymoses extending to the ventral abdomen. Prothrombin time (PT) and activated partial thromboplastin time (aPTT) were prolonged beyond the detectable range. The patient was administered vitamin K1^6^ at 1 mg/kg subcutaneously (SQ). The following day the patient's venipuncture sites continued to bleed and while on a short walk outside the hospital, she collapsed. A new intravenous catheter was placed, and a fresh frozen plasma (FFP) transfusion of unknown volume was administered. A post-transfusion PCV was 24% and total plasma protein (TPP) concentration was 69 g/L (6.9 g/dL; RI 6–8 g/dL). PT and aPTT both remained too prolonged to measure. Abdominal ultrasound by a boarded internist revealed a mildly enlarged liver and single splenic nodule but was otherwise unremarkable. The next day the patient was weak, unwilling to ambulate and pale with bounding pulses. Given the patient's unstable condition, she was subsequently transferred to the authors' institution for further diagnostics and treatment.

On physical examination the patient was obtunded. Temperature, heart rate and respiratory rate were 38.0°C (100.5°F; RI 38.1–39.1°C [100.6–102.4°F]), 150/min and 36/min, respectively. The patient weighed 20.7 kg. Mucous membranes were pale with a capillary refill time of 2s. A grade I/VI left apical systolic heart murmur was auscultated. No arrhythmias were appreciated. Femoral pulses were strong, synchronous and symmetrical. There were increased respiratory sounds bilaterally and mild crackles were auscultated. The mammary tissue was thickened, and some milky discharge was expressed from the caudal glands.

### 2.1. Hematology, biochemistry, and coagulation profile

A 7 French 20 cm central venous catheter^7^ was placed in the left jugular vein due to severe hematomas over both cephalic and saphenous veins. No complications due to the placement of the central venous catheter were noted. PCV was 17% and TPP was 55 g/L (5.5 g/dL). Venous blood gas analysis revealed moderate hyperlactatemia (5.6 mmol/L [50.5 mg/dL]; RI < 2.5 mmol/L [ < 22.5 mg/dL]) and mild hyperventilation (PvCO2 30.5 mmHg; RI 36–44 mmHg) but was otherwise unremarkable. A CBC revealed a severe regenerative anemia (reticulocyte count 145,300/ul; RI 0–60,000/ul), HCT 0.16 L/L [16.1%; RI 40–55%] and moderate thrombocytopenia (platelet count of 63 × 10^9^/L [63,000/uL; RI 150,000–400,000/uL]). Blood smear examination revealed a moderate number of macroplatelets, and no platelet clumps were seen. A chemistry panel showed a mild increase in BUN (13.92 mmol/L [39 mg/dL; RI 11–33 mg/dL] and a mild hypoalbuminemia (30 mg/dL [3.0 g/L; RI 3.4–4.3 g/L]). A coagulation panel showed elevations in PT (26.4s; RI 7.0–9.3 s), aPTT (37.7 s; RI 10.4–12.9 s), D-dimers (346 ng/ml; RI 0–186 ng/ml) and a marked decrease in fibrinogen (< 50 mg/dL; RI 109–311 mg/dL).

### 2.2. Diagnostic imaging

Three-view thoracic radiographs were performed and interpreted by a board-certified radiologist. The cardiac silhouette was within normal limits. The caudal lobar arteries were mildly enlarged compared to the corresponding veins at the proximal aspect but appeared to taper appropriately. There was a diffuse mild bronchial pulmonary pattern. Abdominal ultrasound was also performed by a boarded radiologist and revealed an enlarged, hyperechoic liver with poorly defined nodules. The previously reported splenic nodule was recognized. Ventral gastric margin changes were also noted and interpreted as possible sub-serosal hemorrhage. An echocardiogram was performed by a boarded cardiologist. The quality of the study was poor due to lung interference with imaging. There was mild to moderate right ventricular concentric hypertrophy. No tricuspid regurgitation was appreciated. The main pulmonary artery and in particular the right branch of the main pulmonary artery were dilated. No worms were clearly visualized in the main pulmonary artery. The interventricular septum was mildly flattened in systole. Overall, the findings suggested moderate pulmonary hypertension.

### 2.3. Patient management

The patient was hospitalized in the ICU and administered one unit of type-specific FFP over 1 h and one further unit over the following 2 h (total 14 ml/kg). One unit of type-specific, cross matched packed red blood cells was also administered (7 ml/kg). A constant rate infusion of FFP was then continued at 1 ml/kg/hr. The patient was administered sildenafil^8^ 20 mg (1 mg/kg) PO q 8 hrs and dexamethasone SP^9^ at 0.15 mg/kg IV q 24 hrs. Aminocaproic acid^10^ was administered intravenously at 50 mg/kg over one h followed by a constant rate infusion at 15 mg/kg/hr. A board-certified oncologist assessed the thickened mammary tissue and palpated some firmer masses. Fine needle aspiration was not performed due to the coagulopathy. By the evening the patient was brighter, more active and ate some food. Repeat bloodwork showed a PCV of 18%, TPP 61 g/L (6.1 g/dL), ACT 176 s and lactate 4.0 mmol/L. Overnight, the PCV decreased to 16% with a TPP of 64 g/L (6.4 g/dL) and activated clotting time (ACT) was 999s (RI < 95s). A 25 mg dose of Vitamin K1^11^ PO was administered. A further 150 ml unit of packed red blood cells and 260 ml of FFP were administered over 2 h. Repeat CBC approximately 20 h after admission showed persistent anemia and thrombocytopenia with a HCT of 15.2% and platelet count of 27,000/μl (with evidence of platelet clumping). Despite ongoing administration of FFP, a repeat coagulation panel approximately 17 h after the first assessment showed persistent coagulopathy with a PT of 84.2 s, aPTT > 140 s and fibrinogen < 50 mg/dL. Post-transfusion ACT remained > 999 s with a PCV of 12%. Following discussion with the owners, the patient was euthanized.

### 2.4. Necropsy and histopathological findings

**Figure 1:**
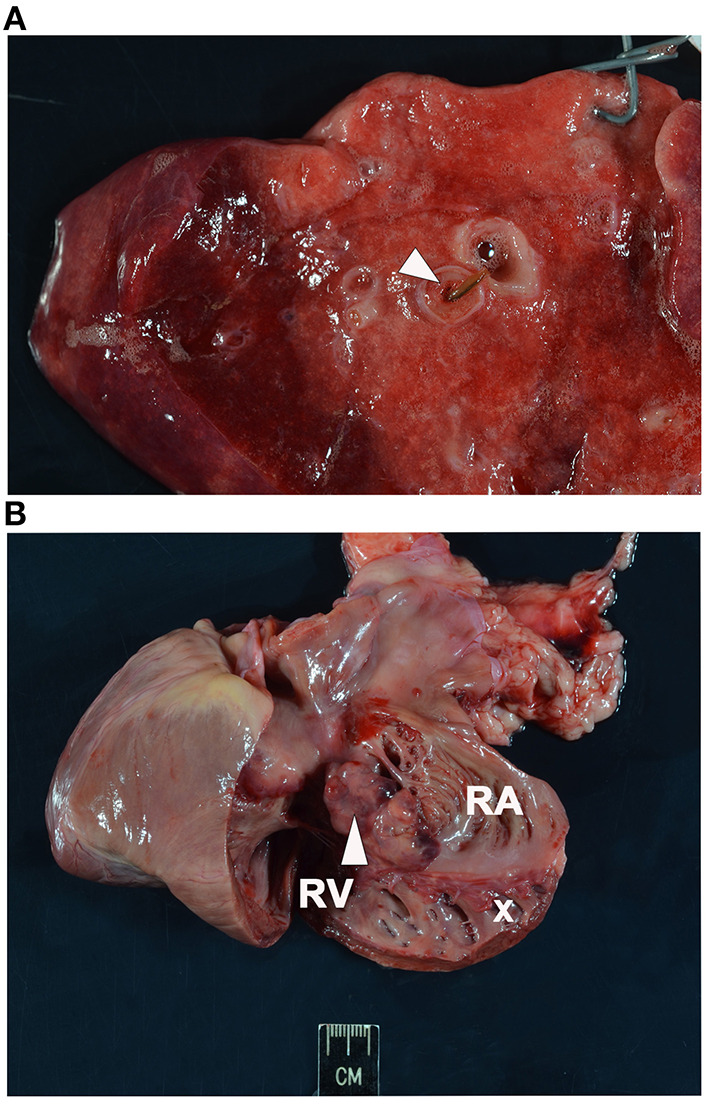
Necropsy images. **(A)** Section of lung showing intraluminal degenerate nematode (arrowhead). **(B)** Section of heart with soft tissue invasion by a lobulated chemodectoma (arrowhead). The right side of the heart is opened with the right ventricle (RV) and dilated right atrium (RA) exposed and the tricuspid valve labeled (x).

Necropsy was performed and confirmed heartworm infection with several adults within the pulmonary artery and right atrium. Within the pulmonary artery and throughout the lungs there was severe, multifocal thrombosis with multifocal endarteritis, severe neo-intimal proliferation, multifocal mural degeneration and fibrosis. There was an invasive chemodectoma (paraganglioma) at the heart base, with invasion into the myocardium of the right atrium and an expansile mass protruding into the chamber of the right ventricle (Figure 1). The mammary gland lesions were benign (adenoma and lobular hyperplasia with secretory activity). Multifocal subcutaneous and organ hemorrhage was apparent throughout the thoracic and abdominal walls as well as the swollen right pelvic limb. Histopathology of the liver revealed vacuolar hepatopathy with associated nodular hyperplasia. The splenic nodule was determined to be a myelolipoma. No hepatic or splenic malignancy was identified.

## 3. Discussion

This case report details the diagnosis and treatment of a dog with severe coagulopathy consistent with DIC following melarsomine treatment for *D. immitis*. The patient in this report had been treated according to current AHS guidelines except that she was administered doxycycline for one week longer than the recommended 30 day course (5).

The pathogenesis of DIC involves initiation of coagulation by tissue factor exposure to circulating blood, increased platelet-vessel wall interaction, impaired regulation of coagulation and defective endogenous fibrinolysis (12). Diagnosis of DIC in people and animals is based on the presence of a primary condition known to be associated with DIC along with a compatible clinical picture (12). A veterinary scoring system for DIC has been proposed and is based on elevated aPTT, PT, D-dimers and decreased fibrinogen with a diagnostic sensitivity and specificity of 90.9 and 90.0% (13). The patient in this report fulfilled all criteria. DIC phenotypes have also been defined according to the associated level of fibrinolytic system activation. Hypofibrinolytic type DIC, characterized by strong coagulation activation and suppressed fibrinolysis, may lead to hemorrhage by development of a consumptive coagulopathy (14). Conversely, enhanced-fibrinolytic DIC is caused by excessive fibrinolysis in which the fibrinolytic pathway is upregulated (12), leading to a severe bleeding diathesis (14). Based on the extent of hemorrhage noted in this patient, a combination of consumptive coagulopathy from dysregulated thrombosis and enhanced fibrinolysis may have contributed to the pathogenesis.

DIC is a recognized complication in heartworm-infected dogs with caval syndrome (3). The patient in this report had echocardiographic findings indicative of pulmonary hypertension and adults were present within the right atrium on necropsy. However, the classic constellation of clinical and echocardiographic signs expected with this syndrome such as hemoglobinuria, cardiovascular collapse and right-sided heart failure were not observed (7).

DIC has previously been reported clinically in heartworm-infected dogs following treatment with parasiticides including thiacetarsamide (the predecessor to melarsomine) (8, 9) and dichlorvos (10). The authors of one report suggested massive tissue factor release due to direct damage from the parasite and secondary to ischemia from thromboembolic disease as a potential inciting cause (9). Some procoagulant effects of thiacetarsamide have also been demonstrated in heartworm-negative dogs, suggesting this drug may have some direct effects on coagulation (15).

DIC is listed as a potential complication (occurring in < 1.5%) of dogs treated with melarsomine dihydrochloride on the drug data sheet (11). Lethargy and some degree of discomfort as recognized in the patient in this report are relatively common following its administration. However, the authors were unable to find any published data on melarsomine-induced coagulopathy in dogs. Melarsomine dihydrochloride is an organic arsenical chemotherapeutic agent. The AHS currently recommends using the three-dose melarsomine protocol for all heartworm-positive dogs to increase the efficacy and safety of treatment (5). The exact mode of action of melarsomine on *D. immitis* is unknown. Adverse reactions observed after treatment with melarsomine may be directly attributable to the medication or may be secondary to worm death or the underlying heartworm disease process (3). If toxicity secondary to melarsomine is suspected, dimercaprol has been reported as an antidote for arsenic toxicity (16). However, dimercaprol does carry a substantial list of side effects and would not have been appropriate in this dog. Ultimately, successful adulticidal treatment in dogs with heartworm disease may inevitably lead to thromboembolism and an intense pro-inflammatory reaction, which conceivably could lead to DIC and/or hyperfibrinolysis (5).

It is also possible that the patient became coagulopathic due to direct effects of the heartworm infection. Pathologic elevations of D-dimers have been demonstrated in dogs with *D. immitis* infection and seem to be highest in microfilaremic dogs (17, 18). It has been suggested that the nematode could use anticoagulant properties of its antigens to control the formation of blood clots in its immediate intravascular habitat as a survival mechanism (19). *D. immitis* excretory/secretory antigens have been shown to stimulate the vascular endothelium to enhance the expression of tissue plasminogen activator and urokinase-type plasminogen activator, to activate fibrinolysis *via* plasminogen activation, to inhibit coagulation factor Xa and to decrease plasminogen activator inhibitor 1 levels (19–21). *Wolbachia*, the endosymbiont bacteria of filarial nematodes, may also have a role in stimulating plasmin generation on its release from dying filarial nematodes (22). However, this patient had been treated with doxycycline prior to adulticide therapy.

A paraneoplastic cause of DIC is also possible in this patient. Hemostatic derangements in cancer patients may develop *via* multiple mechanisms. These include inherent abnormalities of tumor microvasculature, invasion into blood vessels and/or tissue factor expression by neoplastic cells (23).

The dog in this report had undergone resection of a cutaneous HSA 4 months prior to presentation and a chemodectoma was recognized on necropsy. HSA is commonly associated with hemostatic abnormalities including DIC (24). However, cutaneous HSA is typically discrete and surgical excision often curative (25). In addition, solar-induced HSA in a ventral location seems to be inherently less aggressive than dermal HSA of other etiology (26). No evidence of HSA in the skin or other organs was found on gross necropsy or histopathology. The authors could not find a report of DIC resulting from chemodectoma which is generally considered relatively benign with a low metastatic potential (27, 28). The most commonly reported adverse effects associated with chemodectoma include altered cardiovascular function caused by the mass effect or, more commonly, local hemorrhage/effusion into the pericardial space (27, 29). The chemodectoma may also have contributed to the coughing reported in this dog (29). No pericardial effusion or arterial invasion was detected on necropsy in this patient but there was some myocardial infiltration and compression of the right ventricle which could have exacerbated pulmonary hypertension and right ventricular dysfunction.

Antifibrinolytic therapy was administered to the patient in this report in an attempt to enhance clot stability and was initiated due to the severity of bleeding. Ideally viscoelastic testing would have been performed to determine if there was any hyperfibrinolytic component to the hemorrhagic diathesis and to help guide therapy. In dogs with hemorrhage due to *Angiostrongylus vasorum* infection, viscoelastic testing has been utilized to diagnose hypocoagulability and hyperfibrinolysis and to guide therapy with FFP and antifibrinolytic drugs (30, 31). Potential disadvantages to the empirical use of aminocaproic acid include the risk of side effects and the concern that, in general, patients with DIC should not be treated with antifibrinolytic agents. However, in human medicine, patients with DIC that are characterized by a primary hyperfibrinolytic state and who present with severe bleeding may be treated with lysine analogs (32). Overall, antifibrinolytic therapy was considered justified in this patient based on the wide array of described applications and favorable safety profile described in dogs thus far as well as the severity of hemorrhage.

In conclusion, successful adulticide treatment in dogs with significant heartworm disease inevitably leads to thromboembolism and an intense pro-inflammatory reaction which could lead to hyperfibrinolysis and/or DIC. DIC specifically is listed as a potential adverse reaction to melarsomine therapy but is expected to be rare. It is not clear whether DIC directly results from the drug or is secondary to subsequent worm death. Treatment recommendations for hemorrhage associated with DIC include addressing the underlying cause where possible and blood product administration. Antifibrinolytic agents are also indicated in enhanced-fibrinolytic type DIC (32). Sadly, despite all these treatments the coagulopathy in the patient in this report could not be controlled.

## Data availability statement

The original contributions presented in the study are included in the article/supplementary material, further inquiries can be directed to the corresponding author.

## Author contributions

HP and RL contributed toward study design, data collection, and editing. RL is the senior author. KF contributed to editing and data collection. All authors contributed to the article and approved the submitted version.

## Footnotes

^1^SNAP 4dx Plus Test, IDEXX, Westbrook, Maine.

^2^Advantage Multi™ for Dogs (10% imidacloprid + 2.5% moxidectin), Bayer HealthCare, Shawnee, Kansas.

^3^Doxycycline tablets, Ranbaxy Pharmaceuticals, Jacksonville, Florida.

^4^Prednisone tablets, LLOYD Inc., Shenandoah, Iowa.

^5^Melarsomine dihydrochloride, Zoetis Inc., Parsippany, New Jersey.

^6^Vitamin K1 Injectable, VetOne, Sparhawk Laboratories, Inc., Lenexa, Kansas.

^7^Arrow Central venous catheterization kit, Arrow International, Reading, Pennsylvania.

^8^Sildenafil, Pfizer Inc, New York, New York State.

^9^Dexamethasone SP 4 mg/mL, Bimeda US, Oakbrook Terrace, Illinois.

^10^Aminocaproic acid injection, Hospira Inc, Lake Forest, Illinois.

^11^Vitamin K1 tablets, VetOne, Sparhawk Laboratories, Inc., Lenexa, Kansas.

## References

[1] Anvari D Narouei E Daryani A Sarvi S Moosazadeh M Hezarjaribi HZ. The global status of Dirofilaria immitis in dogs: a systematic review and meta-analysis based on published articles. Res Vet Sci. (2020) 131:104–16. 10.1016/j.rvsc.2020.04.002 32330696

[2] Hoch H Strickland K. Canine and feline dirofilariasis: life cycle, pathophysiology, and diagnosis. Compend Contin Educ Vet. (2008) 30:133–40. Available online at: http://vetfolio-vetstreet.s3.amazonaws.com/mmah/b7/cc1763a1484ec8a8facf21a67696dd/filePV_30_03_133.pdf 18409140

[3] Ames MK. Heartworm disease. In: Drobatz KJ, Hopper K, Rozanski EA, Silverstein DC, editors. Textbook of Small Animal Emergency Medicine. Hoboken, NY: John Wiley & Sons, Ltd. (2018) 362–371. 10.1002/9781119028994.ch58

[4] Kramer L Simon F Tamarozzi F Genchi M Bazzocchi C. Is Wolbachia complicating the pathological effects of Dirofilaria immitis infections? Vet Parasitol. (2005) 133:133–6. 10.1016/j.vetpar.2005.04.011 15885912

[5] Available online at: https://www.heartwormsociety.org/images/pdf/2018-AHS-Canine-Guidelines.pdf (accessed November 5, 2022).

[6] Noack S Harrington J Carithers DS Kaminsky R Selzer PM. Heartworm disease – Overview, intervention, and industry perspective. Int J Parasitol. (2021) 16:65–89. 10.1016/j.ijpddr.2021.03.004 34030109 PMC8163879

[7] Strickland KN. Canine and feline caval syndrome. Clin Tech Small Anim Pract. (1998) 13:88–95. 10.1016/S1096-2867(98)80012-1 9753797

[8] Kociba GJ Hathaway JE. Disseminated intravascular coagulation associated with heartworm disease in the dog. J Am Anim Hosp Assoc. (1974) 10:373–8.9753797

[9] Dillon AR Braund KG. Distal polyneuropathy after canine heartworm disease therapy complicated by disseminated intravascular coagulation. J Am Vet Med Assoc. (1982) 181:239–42. 6286583

[10] Barsanti JA Greene CE. Disseminated intravascular coagulation in a heartworm infected dog treated with dichlorvos. Auburn Veterinarian. (1975) 31:110–2.2768039

[11] Available online at: https://www2.zoetisus.com/content/_assets/docs/Petcare/diroban-prescribing-information.pdf (accessed November 5, 2022).

[12] Levi M Sivapalaratnam S. Disseminated intravascular coagulation: an update on pathogenesis and diagnosis. Expert Rev Hematol. (2018) 11:663–72. 10.1080/17474086.2018.1500173 29999440

[13] Wiinberg B Jensen AL Johansson PI Kjelgaard-Hansen M Rozanski E Tranholm M. Development of a model based scoring system for diagnosis of canine disseminated intravascular coagulation with independent assessment of sensitivity and specificity. Veter J. (2010) 185:292–8. 10.1016/j.tvjl.2009.06.003 19586785

[14] Birkbeck R Humm K Cortellini S. A review of hyperfibrinolysis in cats and dogs. J Small Anim Pract. (2019) 60:641–55. 10.1111/jsap.13068 31608455

[15] Boudreaux MK Dillon AR. Platelet function, antithrombin-III activity, and fibrinogen concentration in heartworm-infected and heartworm-negative dogs treated with thiacetarsamide. Am J Vet Res. (1991) 52:1986–91. 1789512

[16] Plumlee KH. Chapter 24 - Pharmaceuticals. In: Plumlee KH, ed. Clinical Veterinary Toxicology. USA: Mosby (2004). p. 282–336. 10.1016/B0-32-301125-X/50027-3

[17] Carretón E Morchón R González-Miguel J Simón F Juste MC Montoya-Alonso JA. Variation of d-dimer values as assessment of pulmonary thromboembolism during adulticide treatment of heartworm disease in dogs. Vet Parasitol. (2013) 195:106–11. 10.1016/j.vetpar.2013.01.005 23384581

[18] Carretón E Morchón R Simón F Juste MC Méndez JC Montoya-Alonso JA. Cardiopulmonary and inflammatory biomarkers in the assessment of the severity of canine dirofilariosis. Vet Parasitol. (2014) 206:43–7. 10.1016/j.vetpar.2014.08.019 25224789

[19] Diosdado A Simón F Morchón R González-Miguel J. Dirofilaria immitis possesses molecules with anticoagulant properties in its excretory/secretory antigens. Parasitology. (2020) 147:559–65. 10.1017/S0031182020000104 31992384 PMC10317642

[20] González-Miguel J Morchón R Mellado I Carretón E Montoya-Alonso JA Simón F. Excretory/secretory antigens from Dirofilaria immitis adult worms interact with the host fibrinolytic system involving the vascular endothelium. Mol Biochem Parasitol. (2012) 181:134–40. 10.1016/j.molbiopara.2011.10.010 22050927

[21] González-Miguel J Morchón R Carretón E Montoya-Alonso JA Simón F. Can the activation of plasminogen/plasmin system of the host by metabolic products of Dirofilaria immitis participate in heartworm disease endarteritis? Parasit Vectors. (2015) 8:194. 10.1186/s13071-015-0799-0 25888952 PMC4391138

[22] Diosdado A Gómez PJ Morchón R Simón F González-Miguel J. Interaction between Wolbachia and the fibrinolytic system as a possible pathological mechanism in cardiopulmonary dirofilariosis. Vet Parasitol. (2017) 247:64–9. 10.1016/j.vetpar.2017.10.001 29080766

[23] Bailey DB. Chapter 5 - Paraneoplastic Syndromes. In: Vail DM, Thamm DH, Liptak JM, ed. Withrow and MacEwen's Small Animal Clinical Oncology (Sixth Edition), St. Louis: W.B. Saunders (2019). p. 98–112. 10.1016/B978-0-323-59496-7.00005-0

[24] Hammer AS Couto CG Swardson C Getzy D. Hemostatic abnormalities in dogs with hemangiosarcoma. J Veter Internal Med. (1991) 5:11–4. 10.1111/j.1939-1676.1991.tb00923.x 2020011

[25] Mullin C Clifford CA. Chapter 34 - Section A: hemangiosarcoma. In: Vail DM, Thamm DH, Liptak JM, ed. Withrow and MacEwen's Small Animal Clinical Oncology (Sixth Edition), St. Louis: W.B. Saunders (2019). p. 787–791.

[26] Szivek A Burns RE Gericota B Affolter VK Kent MS Rodriguez CO. Clinical outcome in 94 cases of dermal haemangiosarcoma in dogs treated with surgical excision: 1993–2007. Vet Comp Oncol. (2012) 10:65–73. 10.1111/j.1476-5829.2011.00282.x 22236371

[27] Treggiari E Pedro B Dukes-McEwan J Gelzer AR Blackwood L A. descriptive review of cardiac tumours in dogs and cats. Vet Comp Oncol. (2017) 15:273–88. 10.1111/vco.12167 26420436

[28] Noszczyk-Nowak A Nowak M Paslawska U Atamaniuk W Nicpon J. Cases with manifestation of chemodectoma diagnosed in dogs in Department of Internal Diseases with Horses, Dogs and Cats Clinic, Veterinary Medicine Faculty, University of Environmental and Life Sciences, Wroclaw, Poland. Acta Vet Scand. (2010) 52:35. 10.1186/1751-0147-52-35 20492718 PMC2886050

[29] Burton JH Stern JA. Chapter 34 Section E: Neoplasia of the heart. In: Vail DM, Thamm DH, Liptak JM, ed. Withrow and MacEwen's Small Animal Clinical Oncology (Sixth Edition), St. Louis: W.B. Saunders (2019). p. 787–791.

[30] Sigrist NE Hofer-Inteeworn N Schefer RJ Kuemmerle-Fraune C Schnyder M Kutter APN. Hyperfibrinolysis and Hypofibrinogenemia Diagnosed With Rotational Thromboelastometry in Dogs Naturally Infected With Angiostrongylus vasorum. J Veter Internal Med. (2017) 31:1091–9. 10.1111/jvim.14723 28480552 PMC5508311

[31] Cole L Barfield D Chan DL Cortellini S. Use of a modified thromboelastography assay for the detection of hyperfibrinolysis in a dog infected with Angiostrongylus vasorum. Veter Record Case Rep. (2018) 6:e000554. 10.1136/vetreccr-2017-000554

[32] Levi M Toh CH Thachil J Watson HG. Guidelines for the diagnosis and management of disseminated intravascular coagulation. Br J Haematol. (2009) 145:24–33. 10.1111/j.1365-2141.2009.07600.x 19222477

